# Role of transcription-regulating kinase CDK8 in colon cancer metastasis

**DOI:** 10.18632/oncotarget.26593

**Published:** 2019-01-18

**Authors:** Jiaxin Liang, Mengqian Chen, Eugenia V. Broude, Igor B. Roninson

**Affiliations:** Department of Drug Discovery and Biomedical Sciences, University of South Carolina, Columbia, SC, USA; Institute of Gene Biology, Russian Academy of Sciences, Moscow, Russian Federation

**Keywords:** CDK8, colon cancer, metastasis, liver, TIMP3

While the best-known members of the cyclin-dependent kinase (CDK) family, such as CDK1, CDK2, CDK4/6, regulate cell cycle progression, many other CDKs play no direct role in cell cycle transitions and instead are involved in transcription or RNA splicing. One of these transcriptional kinases, CDK8 (expressed nearly universally), together with its closely related paralog CDK19 (expressed in a tissue-specific pattern), has a unique function of preferentially regulating transcription of newly activated genes, acting in conjunction with various transcription-initiating factors, such as Wnt/β-catenin [1], serum response network, HIF1A [2], TGFβ/Smad [3], ERα [4], STAT1 and NFκB [5]. As a result, CDK8/19 inhibition has only minor effects on basal transcription but attenuates signal-induced activation of genes that are not expressed in the absence of the signal.

CDK8 has been first linked to cancer when it was identified as an oncogene that is frequently amplified or overexpressed in colon carcinoma [1]. Subsequently, CDK8 has been implicated as a tumor-promoting factor in breast, pancreatic and prostate cancers, melanoma and leukemia [6,7]. CDK8 inhibition was also found to stimulate natural killer cells and to increase innate immunity [8]. Importantly, CDK8 was identified as a mediator of chemotherapy- or radiation-induced expression of genes implicated in cancer metastasis and drug resistance [9]. CDK8/19 has become an actively pursued drug target [6] and the first selective CDK8/19 inhibitor, Senexin B, has recently entered clinical trials. These trials were designed on the basis of preclinical studies where this compound suppressed the growth of estrogen-receptor-positive breast cancers when combined with hormone therapy [4].

Despite the initial reports that CDK8 knockdown by shRNA in colon cancer cells with CDK8 amplification or overexpression inhibited cell proliferation [1], several groups found that CDK8/19 kinase inhibitors have no significant effect on colon cancer cell growth [8,9]. Nevertheless, CDK8 is one of the most frequently amplified genes in clinical colon cancers and elevated CDK8 expression is associated with shorter patient survival. To reconcile these paradoxical observations, we have recently carried out a systematic analysis of the effects of CDK8 knockdown or kinase inhibition on colon cancer cell growth in culture, at different primary tumor sites, as well as in the liver, the primary site of metastasis and the leading cause of colon cancer mortality [10].

In cell culture assays, CDK8/19 kinase inhibition by Senexin B had no effect on short-term growth in three different CDK8-overexpressing human colon cancer cell lines, in two of which CDK8 gene was amplified, whereas only one of the three cell lines showed a significant response in the long-term clonogenic assay. Furthermore, in contrast to previous reports comparing the growth of CDK8-overexpressing colon cancer cells with their derivatives with stable CDK8 knockdown [1], CDK8 knockdown by inducible shRNA expression had no effect on cell growth or colony formation. In subsequent studies, which were conducted primarily in a transplantable murine CT26 colon cancer model, CDK8 knockdown or kinase inhibition were found to have no significant effect on primary tumor growth in mice when the tumor cells were implanted subcutaneously, orthotopically (in the cecum) or in the spleen. In contrast, the growth of tumors that arise in the liver following splenic injection of colon cancer cells, was strongly suppressed by CDK8 knockdown in tumor cells or by treating mice with Senexin B. Selective inhibition of tumor growth in the liver (but not at the primary injection site) was also observed in the CDK8-overexpressing human HCT116 colon cancer cells. Importantly, CDK8/19 inhibitor treatment, when started after liver metastases have been already established, inhibited the growth of metastatic tumors and extended mouse survival.

**Figure 1:**
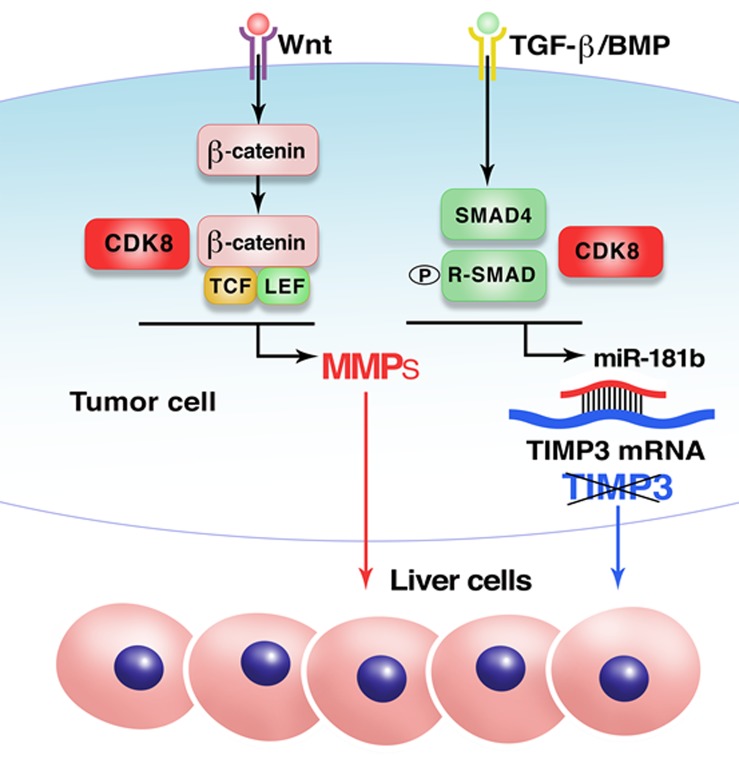
Mechanism of the effect of CDK8 on metastatic growth of colon cancer in the liver

The mechanism of the effect of CDK8 in promoting colon cancer growth in the liver is illustrated in Figure 1. This effect was found to be mediated primarily by the inhibition of expression of extracellular matrix protein TIMP3, which has been implicated in the suppression of invasive growth and angiogenesis. CDK8 suppresses TIMP3 expression by stimulating TGFβ/SMAD-driven transcription of a TIMP3-targeting microRNA, miR-181b, providing a new and potentially general mechanism for negative regulation of gene expression by CDK8. Another effect that contributed to the pro-metastatic activity of CDK8 was the induction of the expression of certain matrix metalloproteinases (MMPs), notably MMP3 in murine and MMP9 in human cells, via the potentiating effect of CDK8 on Wnt/β-catenin-driven transcription. These findings [10] suggest the utility of CDK8 inhibitors for the treatment of colon cancer metastases in the liver and their potential use in other therapeutic settings that involve TGFβ/SMAD or Wnt/β-catenin pathways.
